# Gubi decoction mitigates knee osteoarthritis via promoting chondrocyte autophagy through METTL3‐mediated ATG7 m^6^A methylation

**DOI:** 10.1111/jcmm.70019

**Published:** 2024-08-20

**Authors:** Longkang Cui, Gaobo Shen, Yang Yu, Zheng Yan, Hanbing Zeng, Xiaoang Ye, Kuangying Xu, Chaojin Zhu, Yanan Li, Zhe Shen, Bingbing Zhang, Lianguo Wu

**Affiliations:** ^1^ The Second Clinical College Zhejiang Chinese Medical University Hangzhou China; ^2^ The Second Affiliated Hospital of Zhejiang Chinese Medical University Hangzhou China

**Keywords:** autophagy, chondrocyte, Gubi decoction, knee osteoarthritis, m^6^A modification, METTL3

## Abstract

Knee osteoarthritis (KOA) is a chronic joint disease that significantly affects the health of the elderly. As an herbal remedy, Gubi decoction (GBD) has been traditionally used for the treatment of osteoarthritis‐related syndromes. However, the anti‐KOA efficacy and mechanism of GBD remain unclear. This study aimed to experimentally investigate the anti‐KOA efficacy and the underlying mechanism of GBD. The medial meniscus (DMM) mice model and IL‐1β‐stimulated chondrocytes were, respectively, constructed as in vivo and in vitro models of KOA to evaluate the osteoprotective effect and molecular mechanism of GBD. The UPLC–MS/MS analysis showed that GBD mainly contained pinoresinol diglucoside, rehmannioside D, hesperidin, liquiritin, baohuoside I, glycyrrhizic acid, kaempferol and tangeretin. Animal experiment showed that GBD could alleviate articular cartilage destruction and recover histopathological alterations in DMM mice. In addition, GBD inhibited chondrocyte apoptosis and restored DMM‐induced dysregulated autophagy evidenced by the upregulation of ATG7 and LC3 II/LC3 I but decreased P62 level. Mechanistically, METTL3‐mediated m^6^A modification decreased the expression of ATG7 in DMM mice, as it could be significantly attenuated by GBD. METTL3 overexpression significantly counteracted the protective effect of GBD on chondrocyte autophagy. Further research showed that GBD promoted proteasome‐mediated ubiquitination degradation of METLL3. Our findings suggest that GBD could act as a protective agent against KOA. The protective effect of GBD may result from its promotion on chondrocyte autophagy by suppressing METTL3‐dependent ATG7 m^6^A methylation.

## 1 INTRODUCTION

Osteoarthritis (OA) is a common and degenerative joint disease characterized by the progressive degradation and destruction of articular cartilage. Generally, the knee is considered to be the most prevalent joint affected by osteoarthritis. Knee osteoarthritis (KOA) could lead to functional disability and affect nearly 40% of the aging population worldwide, imposing massive social and economic burdens.^1^ To date, effective treatments for patients with KOA are still insufficient or poorly tolerated. Non‐steroid anti‐inflammatory drugs (NSAIDs) and some anti‐KOA drugs, like diacerein and glucosamine, are only palliative for patients with mild and early KOA.^2^, ^3^ For patients with advanced KOA, arthroplasty remains the only choice.^4^ The pathogenesis of KOA is multifactorial, and the risk factors mainly include genetics, previous trauma, age, obesity and stress, as could provoke articular cartilage degeneration.^5^ Recently, numerous evidence has demonstrated that chondrocyte apoptosis triggers the destruction of cartilage.^6^, ^7^ However, autophagy, a highly conserved degradation system, exhibits a protective effect on damaged chondrocytes and alleviates the progression of KOA.^8^ Actually, inhibiting the apoptosis of chondrocytes by autophagy activation has attracted much attention of scholar.

Autophagy is an important metabolic pathway to modulate inflammation and maintain stability of intracellular environment in the body.^9^ The impairment of autophagic activity could down‐regulate clearance efficiency, eventually facilitate cell degeneration or even apoptosis.^10^ Recent studies have shown that, under the pathogenic conditions of KOA, the level of autophagy in chondrocytes decreases and is closely associated with the increased severity of KOA.^11^ Lian et al. reported that microRNA‐128a repressed chondrocyte autophagy and exacerbates KOA by disrupting ATG12.^12^ Activation of autophagy could protect chondrocytes from degradation and apoptosis, and thus effectively alleviate the development of KOA. For example, restoring autophagy by mechanical loading or targeting TNFR1‐associated death domain protein were proved to effectively retard OA pathological progression and mitigate OA symptoms at both early and late stages.^13^, ^14^ Rapamycin, a selective mTOR inhibitor, was also reported to protect chondrocytes against IL‐18‐induced apoptosis and ameliorate OA via promoting autophagy.^15^ These findings suggest that activating autophagy of articular chondrocytes may be a promising strategy for KOA prevention.

In clinical practice, due to the advantages of remarkable curative effect, minimal side effects and low cost, herbal medicine has presented tremendous promise in OA treatment when compared to Western medicine.^16^ Gubi decoction (GBD) is a classical prescription composing of *Rehmannia glutinosa* Libosch. (RG), *Eucommia ulmoides* Oliv. (EU), *Epimedium brevicornu* Maxim.(EB), *Achyranthes bidentata* Bl.(AB), *Citrus reticulata* Blanco. (CR), *Buddleja officinalis* Maxim. (BO), *Carthamus tinctorius* L. (CT) and *Glycyrrhiza uralensis* Fisch. (GU) (The plant names have been checked with http://www.theplantlist.org/).^17^ GBD has been traditionally applied in clinical practice to treat osteoporosis, KOA and other ageing diseases for several decades.^18^ Modern pharmacological studies showed that the effective components in GBD obviously protected against OA. For example, catalpol could prevent chondrocyte apoptotic level triggered by IL‐1ß, and inhibit cartilage degeneration in the knee joint of OA rat.^19^ Moreover, *Achyranthes bidentata* polysaccharides, the active component of Achyranthis Bidentatae Radix, was proved to promote chondrocyte proliferation by activating the Wnt/β‐catenin signalling pathway.^20^ Clinical investigations showed that GBD can effectively relieve pain and improve knee joint function in patients with OA, and has a significant inhibitory effect on serum TNF‐α and IL‐β level.^21^ However, the specific mechanism that GBD against KOA is still unclear and remains to be fully elucidated. In the present study, we aimed to investigate the molecular mechanism underlying GBD in treating KOA. We constructed the mice model of KOA and cultivated IL‐1β‐induced chondrocytes to explore whether the protective effects of GBD was mediated by inhibiting chondrocyte autophagy.

## 2 MATERIALS AND METHODS

### 2.1 Herbal preparation

RG, EU, EB, AB, CR, BO, CT and GU were purchased from Tong Ren Tang (Hanzhou, China). Voucher specimens of the above herbs were stored in the herbarium of Zhejiang Chinese Medical University, Hanzhou, China, labelled as RG20210116, EU20210516, EB20210116, AB20210116, CR20210116, BO20210116, CT20210116 and GU20210116. GBD extract was prepared as follows: RG 15 g, EU 15 g, EB 15 g, AB 15 g, CR 15 g, BO 10 g, CT 10 g and GU 9 g, were mixed. Crude drugs were boiled in 10 volume (*v*/*w*) of distilled water for 1 h. Following filtration, the residue was further extracted in 8 volume of water for 1 h. Combined filtrate was concentrated under reduced pressure to 1 g crude drug/mL.

### 2.2 Preparation of medicine serum

Adult SD male rats were randomly divided into four groups (*n* = 6/group): drug‐free (‘blank’) serum group, low‐dose GBD serum group (0.125 g/kg), middle‐dose GBD serum group (0.25 g/kg), high‐dose GBD serum group (0.5 g/kg). Rats in GBD serum groups were administered with GBD extract intragastrically for consecutive 3 days. On the morning of the 4th day, the abdominal aortic blood were collected and then centrifuged at 3000 rpm for 15 min. The plasma was inactivated by water bath (56°C, 30 min), filtered using 0.22 μm membrane, and stored at −20°C for in vitro study.

### 2.3 Animal models

Sixty C57BL/6 male mice (10–12 weeks old) were purchased from Changzhou Kavins Experimental Animal Co., LTD. The mice were housed in the Experimental Animal Center of Zhejiang University of Chinese Medicine, and acclimated for 1 week prior to the experiment. The animal experiment was approved by the Experimental Animal Ethics Committee of Zhejiang University of Chinese Medicine (no: IACUC‐20210125‐08). Mice were randomly grouped into five groups (*n* = 10): sham; model; model + low‐dose GBD (8.74 g/kg/day); model + middle‐dose GBD (17.48 g/kg/day); model + high‐dose GBD (34.96 g/kg/day); model + diclofenac (10 mg/kg/day). Except the sham operation group, the medial meniscus (DMM) model was established in the other four groups by microsurgery for transecting the medial meniscotibial ligament. Mice in the sham operation group and the DMM model group received normal saline by intra‐gastric gavage. Mice in GBD‐treated groups received oral gavage of different doses of GBD for 8 weeks. Mice in the positive group was treated with 10 mg/kg/day diclofenac by intragastrical administration.

### 2.4 Histological evaluation

At the end of the experiment, the tibia and femur tissues of mice were separated for general histological evaluation, including H&E staining and Safranin‐O staining. The OARSI score was applied to estimate the severity of KOA.

### 2.5 Quantitative micro‐CT evaluation

In order to examine the effects of GBD on bone remodelling, quantitative evaluations of the tibial subchondral bone was performed using micro‐CT (μCT) equipment (PerkinElmer). Micro‐CT scans were performed with the following instrument settings: x‐ray voltage, 90 kV; tube current, 88 μA; voxel size, 90 μm; exposure time, 14 min; continuous (non‐stepping) rotation.

### 2.6 Immunohistochemical (IHC) staining

The cartilage sections of knee joint were incubated with primary antibodies against LC3 at 4°C for overnight, and then incubated with goat anti‐mouse/rabbit HRP‐conjugated secondary antibody. Subsequently, sections were processed with 3,3'‐diaminobenzidine horseradish peroxidase colour development kit (Beyotime, Nanjing, China) prior to counterstaining with haematoxylin. Positively stained cells were visualized using light microscope and quantified using ImageJ software.

### 2.7 Immunofluorescence (IF) staining

For IF staining, the slides from each treatment group were blocked with 5% BSA and then incubated with primary antibodies against MMP13 (Abcam, Cambridge, UK), LC3 (Abcam, Cambridge, UK) and ATG7 (Invitrogen, Carlsbad, USA) overnight at 4°C. The next day, the slices were incubated with a fluorescein‐conjugated secondary antibody (Proteintech, Wuhan, China), and then the nuclei were stained with DAPI (Santa Cruz, USA). A fluorescence microscope (Zeiss, Germany) was used to acquire the images.

### 2.8 TdT‐mediated dUTP Nick‐End Labelling (TUNEL)

The TUNEL Apoptosis Detection Kit (Abbkine Scientific Co., Ltd., Wuhan, China) was applied to evaluate chondrocyte apoptosis in sections. The slides were fixed and permeabilized with 0.1% Triton X‐100, and then incubated with reagent mixture. The following day, the nuclei were stained with DAPI (Santa Cruz, USA), and then observed under an inverted fluorescence microscope (Olympus, Tokyo, Japan).

### 2.9 Chondrocytes isolation and culture

Six‐week‐old SD rats were killed to harvest chondrocytes. The cartilage from both knees and hips were collected under sterile conditions. After that, the cartilage were cut into small pieces, washed and digested with 0.25% trypsin–EDTA solution and 0.2% type II collagenase. Next, the released chondrocytes were harvested and passaged when they reached 80%–90% confluency. Cells at passage 2 and 3 were used for the experiments.

### 2.10 Cell viability assay

The methyl thiazol tetrazolium (MTT) assay was used to determine cell viability following IL‐1β (10 ng/mL) and GBD treatment. Briefly, rat chondrocytes (1 × 10^4^/well) were seeded in 96‐well plates for 24 h. The cells were then treated with IL‐1β or GBD‐containing serum, MTT assay was performed routinely. The optical density value was measured with a microplate reader at 570 nm.

### 2.11 qRT‐PCR and western blot

Total RNA and whole protein extraction of cartilage tissue or chondrocytes were respectively prepared for RT‐PCR and western blot analysis. The primer sequences for qRT‐PCR in this study were as follows: rat ATG7, forward: 5'‐GAGAGCCGATGGCTTCCTAC‐3', reverse: 5'‐CCTGGAGCCACCACATCA TT‐3'; mouse ATG7, forward: 5'‐GCCAACTCCACACTGCTTTC‐3', reverse: 5'‐TCTTCTGGGTCAGTTCGTGC‐3'; Sox9, forward: 5'‐AAGTTGATCTGAAGCG AGAG‐3', reverse: 5'‐TCTCAATGTTGGAGATGACG‐3'; MMP13, forward: 5'‐CATATGAACATCCATCCCGT‐3'; reverse: 5'‐TGTCATAACCATTCAGAGCC‐3'; Runx2, forward: 5'‐CTCCGCTGTTATGAAAAACC‐3', reverse: 5'‐GGAGGATTTGTGAAGACTGT‐3'; TNF‐α, forward: 5'‐ATGAGAAGTTCCCAAATGGC‐3', reverse: 5'‐CTTGGTGGTTTGTGAGTGTG‐3'; IL‐6, forward: 5'‐GTCTTCTGGAGTACCATAGC‐3', reverse: 5'‐TGACTCCAGCTTATCTCTTG‐3'; IL‐1β, 5'‐TGCCACCTTTTGACAGTGATG‐3', reverse: 5'‐GAGATTTGAAGCTGGATGCTC‐3'; and GAPDH, forward: 5'‐AGTGTTTCCTCGTCCCGTAG‐3', reverse: 5'‐GAAGGGGTCGTTGATGGCAA‐3'. Primary antibodies for western blot in this study were anti‐MMP13 (Abcam; ab39012); anti‐LC3(I/II) (CST; 11972); anti‐ATG7 (CST; 8558), anti‐P62 (Abcam; ab109012), anti‐METTL3 (Abcam; ab195352) and anti‐GAPDH (Abcam; ab109012). Secondary antibody was anti‐rabbit IgG (H + L) (CST, 14708).

### 2.12 Flow cytometry analysis

Chondrocytes were seeded onto glass coverslips for overnight. The cells were then stimulated with IL‐1β (10 ng/mL) for 1 h following GBD treatment for 24 h. After that, apoptosis analysis was conducted using Annexin V‐FITC/PI Apoptosis Detection Kit (Keygen Biotech, Jiangsu, China) according to the manufacturer's instructions. The data were detected with a flow cytometry.

### 2.13 m^6^A quantification

The global m^6^A levels of mRNA were determined with an m^6^A RNA Methylation Assay Kit (Colorimetric) following the manufacture's protocol. The absorbance was measured at 450 nm, and the total m^6^A content was calculated based on the standard curve.

### 2.14 MeRIP‐qPCR

Total RNA was obtained and fragmented. A part of the fragmented RNA was saved as input, and the rest RNA sample was immunoprecipitated with beads conjugated by m^6^A‐antibody, and then eluted. The m^6^A immunoprecipitated samples were further analysed using qPCR. The relative enrichment of m^6^A was normalized to the input.

### 2.15 RNA stability assay

Chondrocytes were seeded in six‐well plates overnight. After GBD pre‐treatment, cells were treated with actinomycin D (5 μg/mL, HY‐17559, MedChemExpress) for 0, 20, 40 and 60 min before collection. Total RNAs were isolated, and the half‐life of mRNA was detected by qPCR.

### 2.16 METTL3 overexpression

To METTL3 overexpression, lentivirus containing METTL3‐overexpressing fragment were transfected into chondrocytes using Lipofectamine3000 (Invitrogen). After transfection for 24 h, cells were harvested for subsequent experimental measurement. The scramble shRNA was transfected as a negative control.

### 2.17 Statistical analyses

All data are presented as the means ± SEM. A Student's *t*‐test (two‐tails) was applied for comparison between two groups, while one‐way anova was employed for comparisons involving multiple groups. Differences were considered statistically significant when *p* < 0.05.

## 3 RESULTS

### 3.1 Identification of the compounds in GBD extract by LC‐MS/MS

**FIGURE 1:**
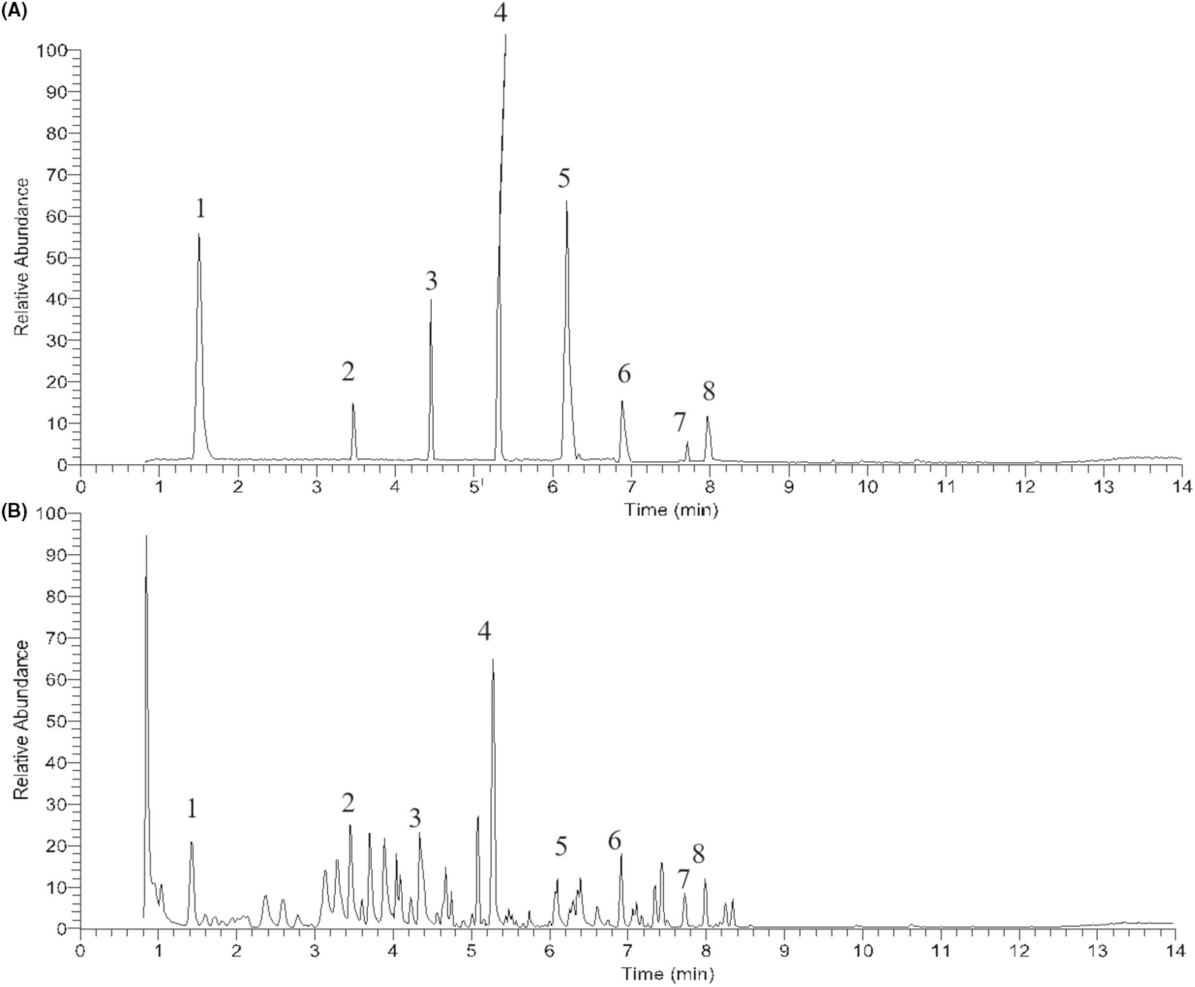
Representative LC‐MS/MS chromatograms of GBD in negative ion mode. (A) Standard compounds. (B) GBD extract.

Firstly, the major chemical ingredients GBD extract were characterized using LC‐MS/MS. The representative total ion chromatograms of standard compounds and GBD extract in negative ion mode were shown as Figure 1A,B. According to measured molecular weight, fragment ion, elemental analysis, and compared with standard chemicals, eight compounds in GBD extract were definitely identified to be pinoresinol diglucoside (peak 1), rehmannioside D (peak 2), hesperidin (peak 3), liquiritin (peak 4), baohuoside I (peak 5), glycyrrhizic acid (peak 6), kaempferol (peak 7) and tangeretin (peak 8).

### 3.2 GBD effectively prevented progression of KOA in DMM mice

**FIGURE 2:**
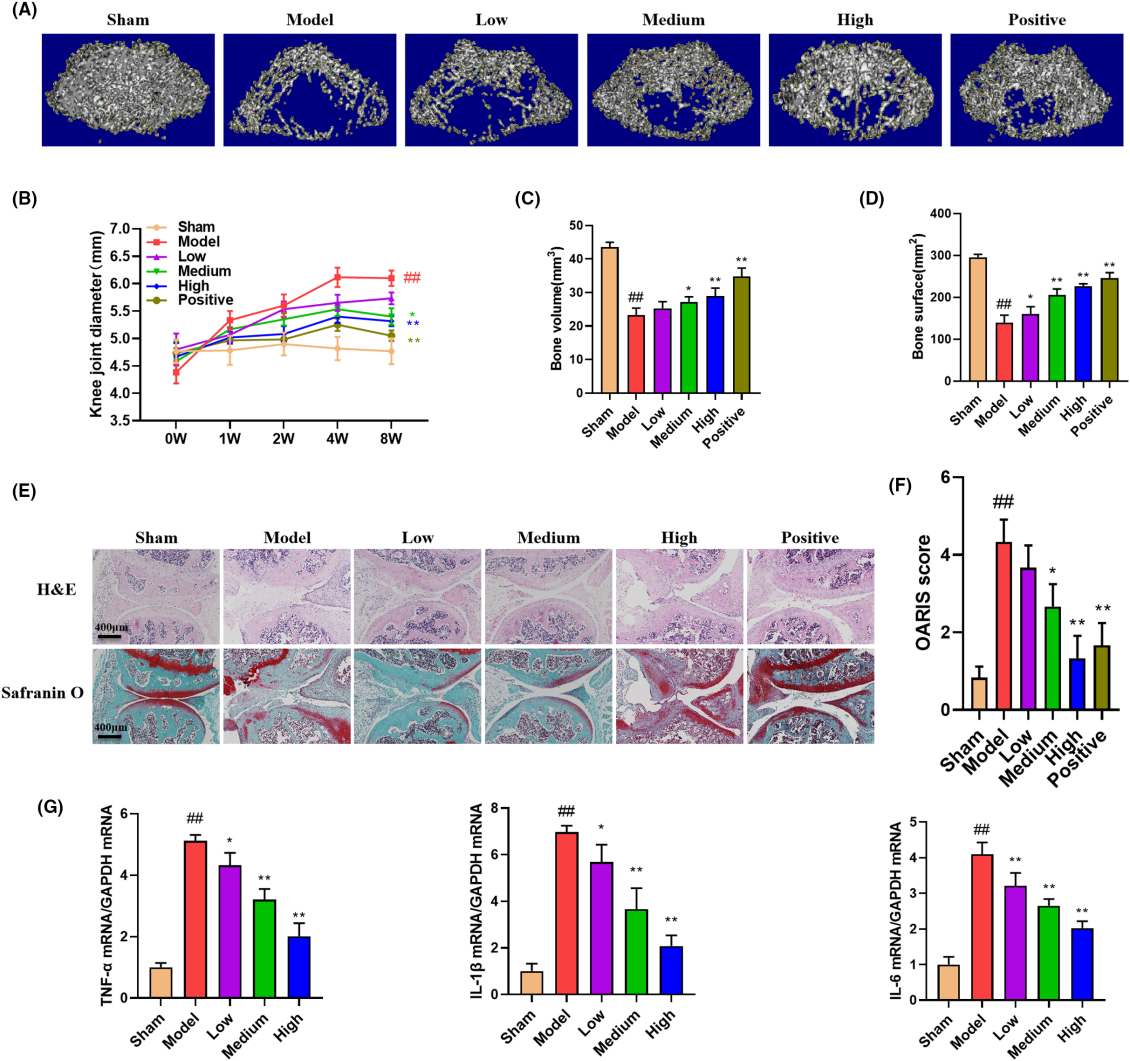
The protective effect of GBD against DMM‐induced knee osteoarthritis. (A) Representative micro‐CT images of subchondral bone of mice. (B–D) Knee joint diameter, bone volume and bone surface of mice from different groups. (E) H&E staining and safranin‐O/fast green staining of cartilage areas. (F) OARSI score. (G) TNF‐α, IL‐1β and IL‐6 mRNA levels in articular cartilage. ^##^ *p* < 0.01 versus control group; *p < 0.05, **p < 0.01 versus model group.

To determine the efficacy of GBD on development of KOA, the changes in microstructure of the subchondral bone was evaluated by 3‐D reconstruction of subchondral bone using quantitative micro‐CT. The results showed that GBD could protect the subchondral bone of DMM mice in a dose‐dependent manner (Figure 2A). GBD‐treated group showed decreased knee joint diameter, increased bone volume and bone surface when compared with the model group (Figure 2B–D).

To elevate the therapeutic effect of GBD on cartilage damage in KOA, the joint of mice was excised, and H&E staining as well as Safranin‐O fast green staining were conducted. As seen in Figure 2E, mice treated with GBD exhibited decreased width and proteoglycan of cartilage component in comparison to model group. Furthermore, GBD decreased cartilage degradation of the knee joint. Consistent with the observed histopathological alterations in the cartilage of mice, GBD significantly reduced cartilage OARSI score elevated by DMM (Figure 2F). Moreover, GBD inhibited the mRNA levels of inflammatory cytokines including TNF‐α, IL6, and IL‐1β in articular cartilage (Figure 2G). Together, these experimental results indicated that GBD could effectively prevented progression of KOA by reducing bone and cartilage damage.

### 3.3 GBD ameliorated cartilage degeneration and inhibited chondrocyte apoptosis

**FIGURE 3:**
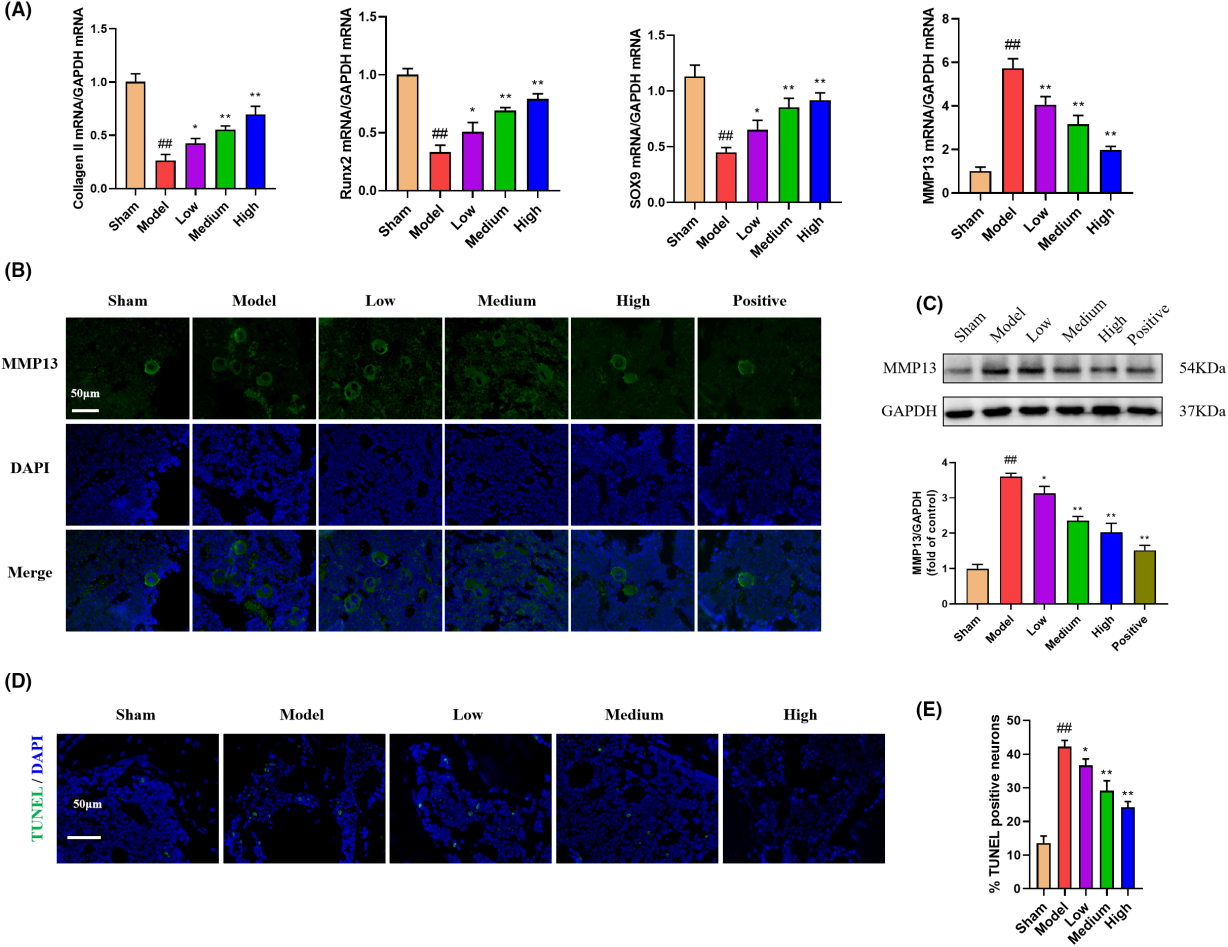
GBD ameliorated cartilage degeneration and inhibited chondrocyte apoptosis. (A) The mRNA expression of collagen II, Runx2 and SOX9 in articular cartilage. (B) Immunohistochemistry assay of MMP13 protein. (C) Western blot images and quantitative analysis of MMP13 in cartilage tissue. (D) Apoptotic cells in cartilage tissue. (E) Graph shows the number of TUNEL‐positive cells. ^##^ *p* < 0.01 versus control group; *p < 0.05, **p < 0.01versus model group.

Articular cartilage is a connective tissue that mainly composed of chondrocytes and extracellular matrix (ECM). We demonstrated the protective effect of GBD on cartilage destruction in DMM mice. Next, we further examined whether GBD affected ECM degradation and chondrocytes apoptosis. Cartilage synthesis metabolism indicators such as collagen II, Runx2, SOX9 and MMP13 were determined by qRT‐PCR. It was found that GBD could promote the mRNA expression of collagen II, Runx2 and SOX9, but inhibited the mRNA level of MMP13 (Figure 3A). MMP‐13 is a major metalloproteinase that involved in the cleavage of type II collagen and cartilage degradation. In our study, different methods were applied to detect the expression of MMP‐13. Results from IF staining and western blot analysis both showed that high expression of MMP13 was induced after DMM surgery (Figure 3B,C). Treatment with GBD markedly reduced MMP13 levels in the cartilage tissue of DMM mice (Figure 3B,C). Additionally, TUNEL staining (in green) were conducted to assess the degree of chondrocyte apoptosis. We found that the number of TUNEL‐positive cells significantly increased in the DMM mice but reduced after GBD treatment (Figure 3D,E).

### 3.4 GBD restored DMM‐induced dysregulated autophagy in cartilage tissue of mice

**FIGURE 4:**
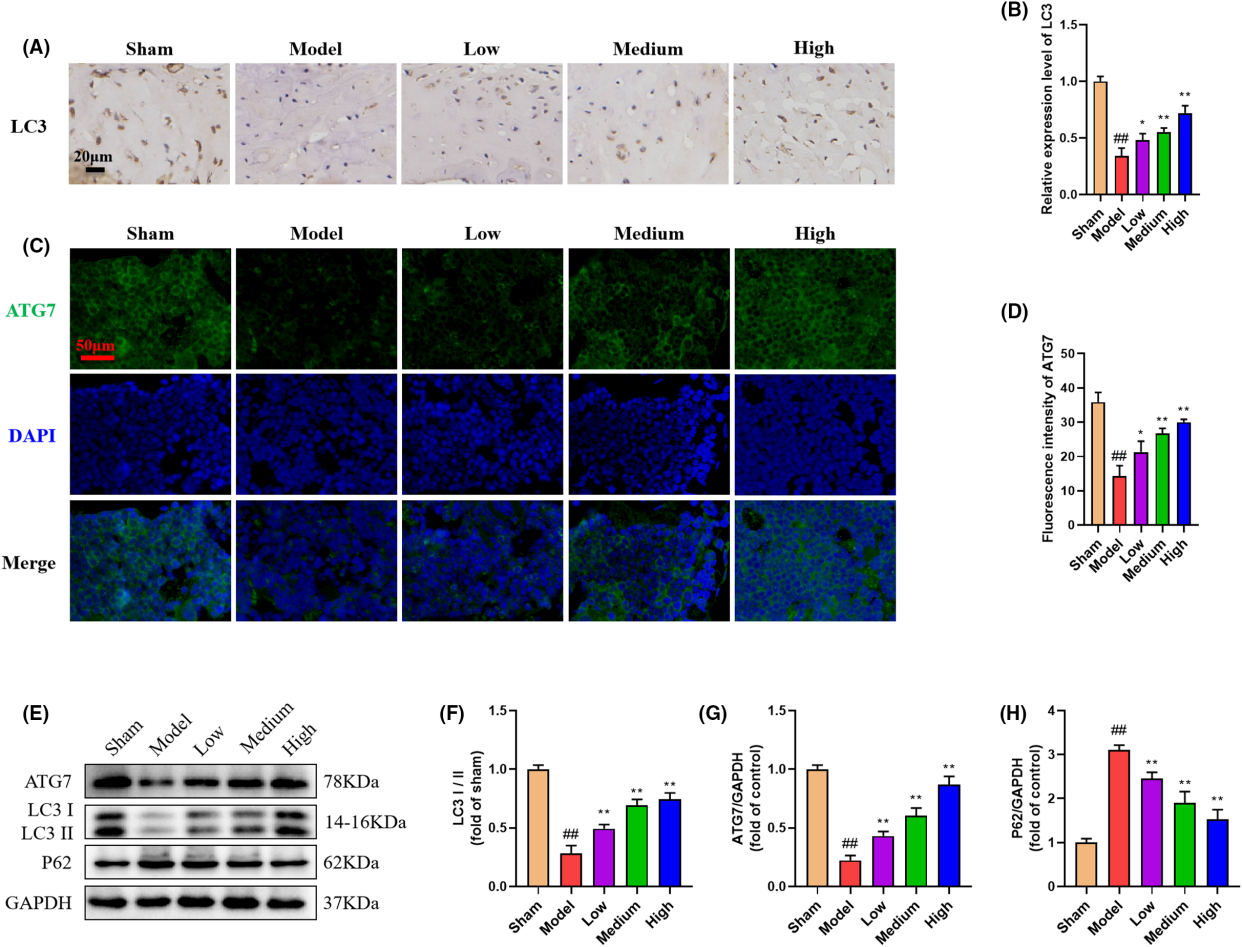
GBD restored DMM‐induced dysregulated autophagy in cartilage tissue of mice. (A, B) Immunohistochemistry and quantification of LC3 positive cells. (C, D) Immunohistochemistry assay of ATG7 protein. (E) Western blot images of ATG7, LC3I, LC3II and P62 in cartilage tissue. (F–H) Quantitative analysis of western blot assay in indicated groups. ^##^ *p* < 0.01 versus control group; *p < 0.05, **p < 0.01 versus model group.

Recently, several evidences have demonstrated that chondrocyte autophagy play vital roles in KOA pathogenesisc.^22^, ^23^, ^24^ Autophagy deficiency would exacerbate cell apoptosis. Therefore, we investigated the regulation effect of GBD on chondrocyte autophagy in this process. As expected, by applying immunohistochemistry and IF analysis, DMM surgery significantly inhibited the expression of autophagy markers including LC3II (Figure 4A,B) and Atg7 (Figure 4C,D). Moreover, western blot analysis also confirmed the degradation of autophagy markers in model group. GBD treatment could enhance the expression of Atg7 and LC3 II and decreased P62 levels in DMM mice (Figure 4E–H). These results indicated that the antiapoptotic activity of GBD in chondrocyte might be associated with its positive regulation of autophagy.

### 3.5 GBD repaired chondrocyte autophagy by suppressing ATG7 m^6^A modification

**FIGURE 5:**
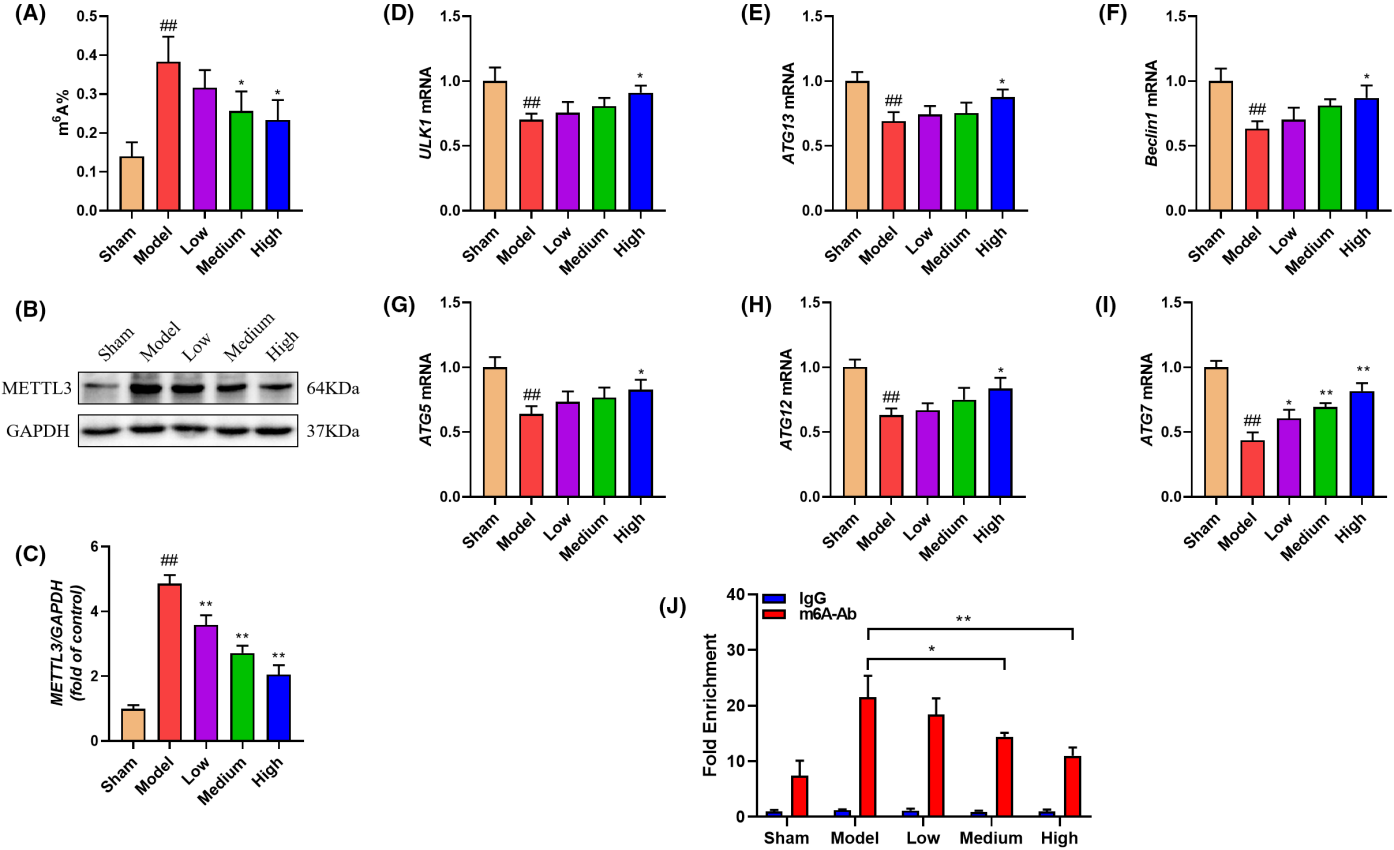
GBD suppressed ATG7 m^6^A modification in vivo. (A) The m^6^A level of RNA in each group (*n* = 3). (B) Western blot image of METTL3 in cartilage tissue. (C) Quantitative analysis of western blot assay in indicated groups. (D–I) ULK1, ATG13, Beclin1, ATG5, ATG12 and ATG7 mRNA detected by qPCR. (J) The effect of GBD on the m^6^A modification of ATG7 was detected by MeRIP‐PCR. ^##^ *p* < 0.01 versus control group; *p < 0.05, **p < 0.01 versus model group.

ATG7 is an autophagy effector enzyme that regulate the cell apoptosis. N6‐methyladenosine (m^6^A) is an important and reversible RNA methylation. A recent evidence has suggest that Mettl3‐mediated m^6^A could control autophagy by targeting ATG7 and then accelerate the progression of KOA.^25^ Since GBD enhanced the expression of ATG7 and restored DMM‐induced dysregulated autophagy, we wonder whether METTL3‐mediated m^6^A is involved in the regulation of GBD on ATG7 expression. The m6A level of RNA was determined by using an m6A RNA methylation assay kit, and METTL3 expression was detected using western blot. The results indicated that the level of m6A in the DMM mice was much higher than in the sham group (Figure 5A), and the level of METTL3 expression also profoundly increased (Figure 5B,C). GBD could markedly inhibit the level of m6A as well as METTL3 expression. To verify the role of METTL3‐mediated m^6^A modification in chondrocyte autophagy, we further examined the mRNA levels of ULK1, ATG13, Beclin1, ATG5, ATG12 and ATG7 by QPCR (Figure 5D–I). Consistent with the ATG7 protein level, ATG7 mRNA were significantly reduced by DMM surgery, and upregulated after GBD treatment (Figure 5I). Furthermore, MeRIP‐qPCR indicated that DMM augmented the m^6^A modification of ATG7 in the model group, whereas GBD administration attenuated the level (Figure 5J). Together, METTL3‐mediated ATG7 m^6^A modification might contribute to the enhancement of GBD on chondrocyte autophagy.

### 3.6 GBD inhibited chondrocyte apoptosis and attenuated autophagy deficiency in vitro

**FIGURE 6:**
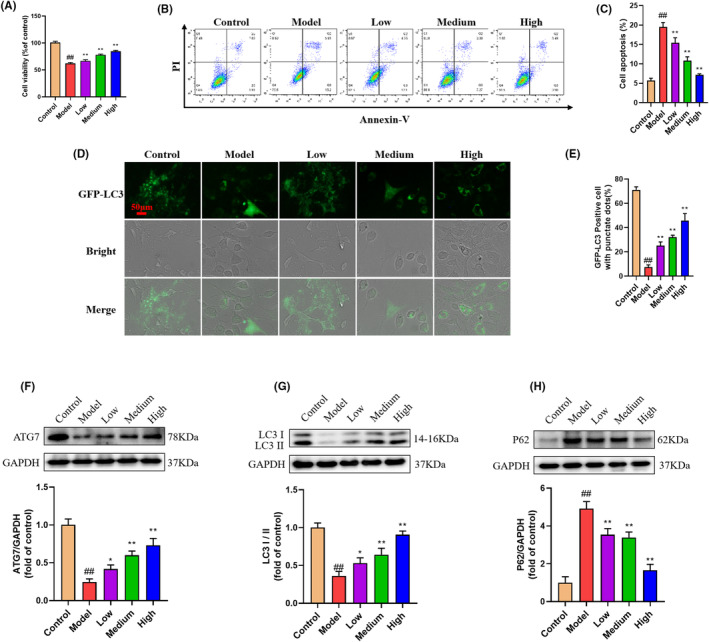
GBD inhibited chondrocyte apoptosis and attenuated autophagy deficiency in IL‐1β‐induced chondrocytes. (A) Cell viability evaluated by MTT. (B, C) Chondrocyte apoptosis detected by flow cytometry. (D, E) Representative images and quantification of GFP‐LC3 in indicated groups. (F) Representative western blot of ATG7 and quantitative analysis data. (G) Representative western blot of LC3I, LC3II and quantitative analysis data. (H) Representative western blot of P62 and quantitative analysis data. ^##^ *p* < 0.01 versus control group; *p < 0.05, **p < 0.01 versus model group.

The chondroprotective effects of GBD were further instigated in isolated rat chondrocytes in vitro. MTT assay and Annexin‐V/PI flow cytometry results showed GBD treatment could significantly improve cell viability and inhibited IL‐1β (10 ng/mL)‐induced chondrocyte apoptosis (Figure 6A,B). Next, IF staining and Western blot suggested that the addition of IL‐1β significantly reduced the autophagy proteins LC3, ATG7, and the LC3II/LC3I ratio, accompanied by enhanced expression of p62 in chondrocyte cells (Figure 6D–H). However, the cells treated GBD showed increased incidence of autophagy compared with the IL‐1β‐stimulated cells (*p* < 0.01). To determine whether the protective effect of GBD on chondrocyte is due to its activation of autophagy, we examined the effects of pharmacological inhibition by 50 nM chloroquine on GBD‐mediated beneficial regulation on chondrocyte apoptosis. The results showed that autophagy inhibition by chloroquine significantly reduced the anti‐apoptotic effect of GBD in chondrocyte cells (Figure S1).

### 3.7 GBD exerted chondroprotective effect by inhibiting METTL3‐mediated ATG7 m^6^A modification in vitro

**FIGURE 7:**
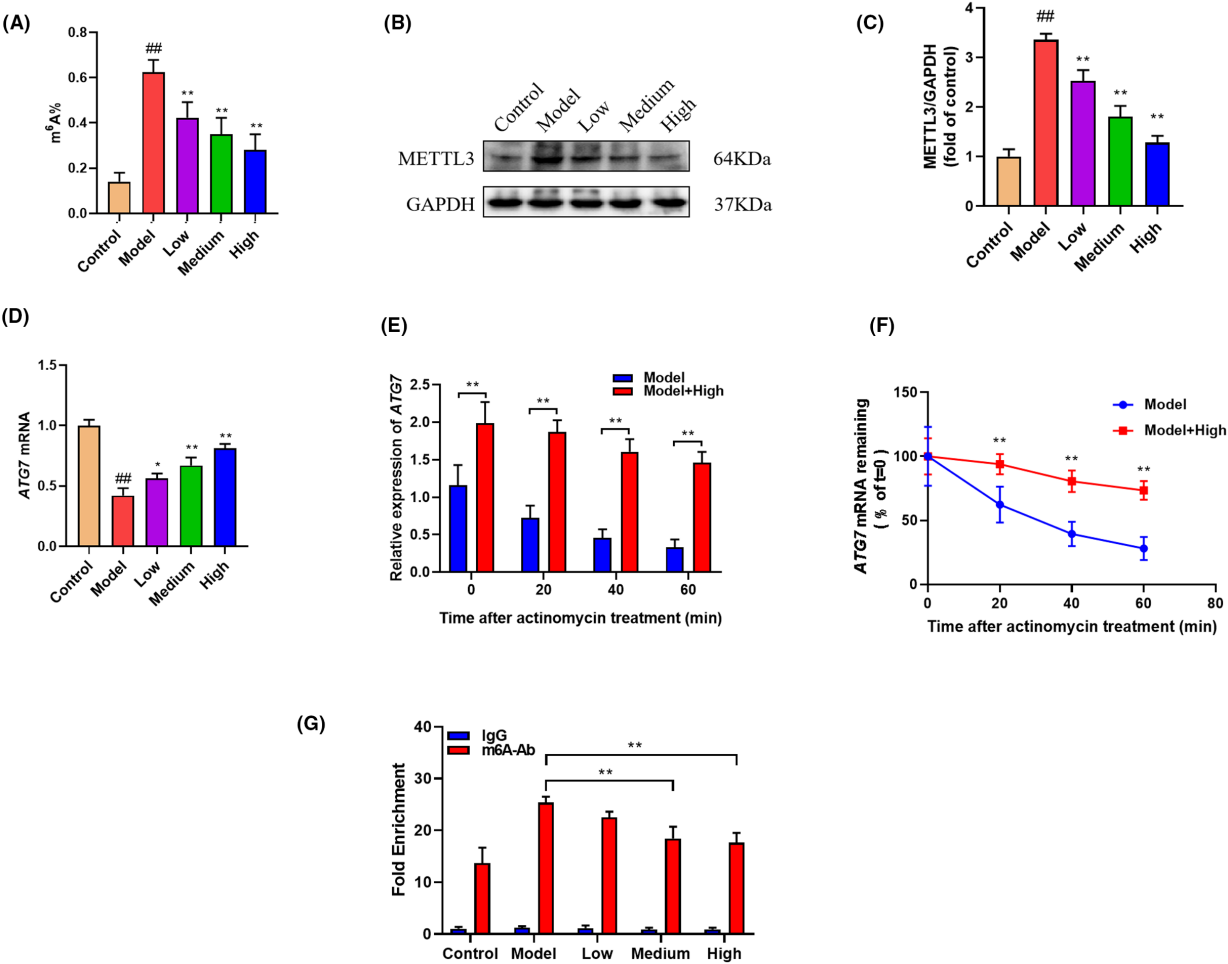
GBD inhibited METTL3‐mediated ATG7 m^6^A modification in vitro. (A) The m^6^A level of RNA in each group. (B) Western blot image of METTL3 in rat chondrocytes. (C) Quantitative analysis of western blot assay in indicated groups. (D) ATG7 mRNA detected by qPCR. (E) The effect of GBD on the RNA stability of ATG7. (F) The effect of GBD on the half‐life of ATG7 mRNA. (G) m^6^A modification of ATG7 detected by MeRIP‐PCR. ^##^ *p* < 0.01 versus control group; *p < 0.05, **p < 0.01 versus model group.

**FIGURE 8:**
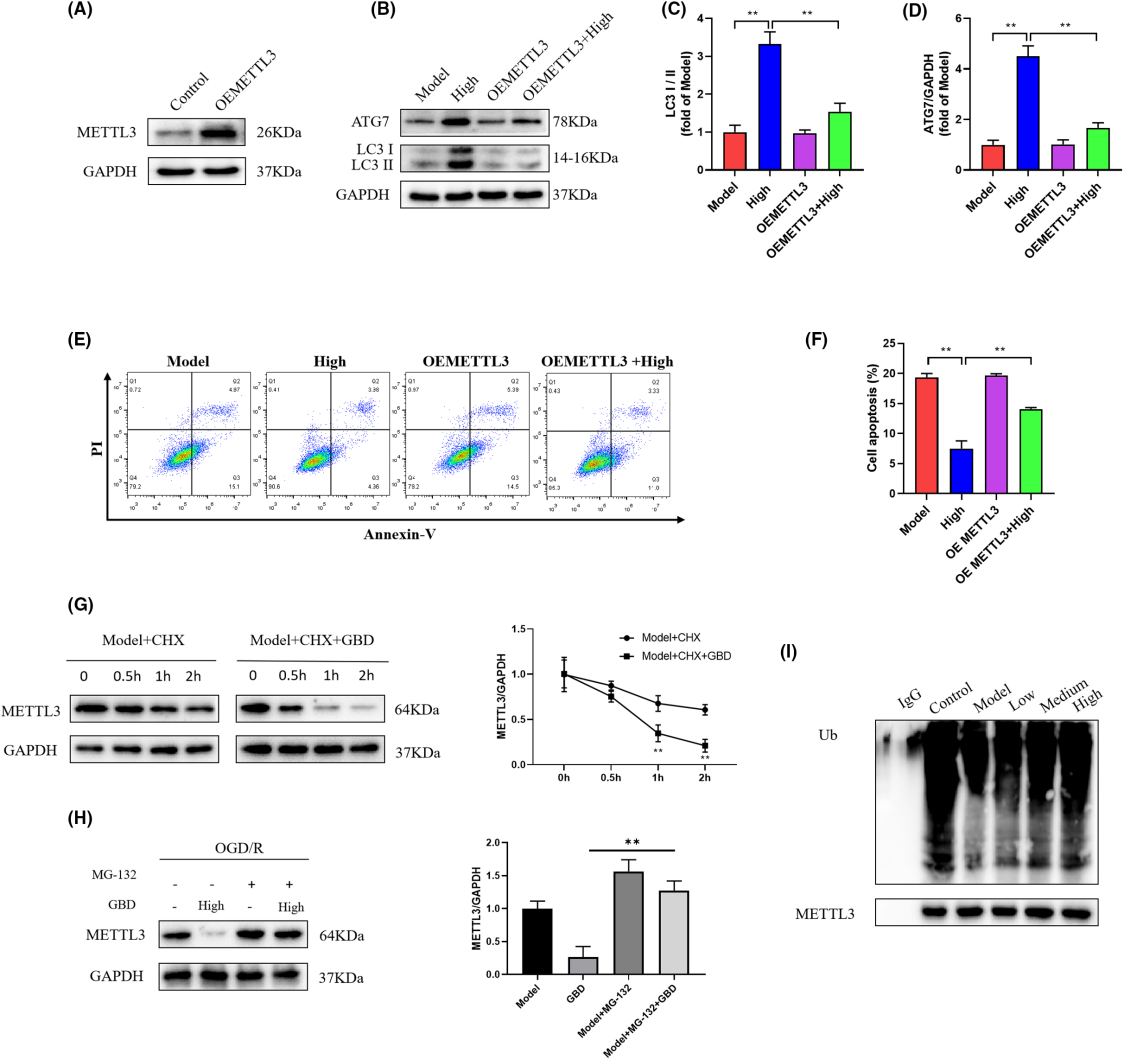
METTL3 overexpression significantly counteracted the protective effect of GBD on chondrocyte apoptosis and autophagy. (A) Western blot analysis of METTL3 expression in indicated groups. (B–D) Expression of LC3I, LC3II and ATG7 in control and METTL3‐overexpressed chondrocyte. (E, F) Cell apoptosis detected by flow cytometry in METTL3‐overexpressed chondrocyte. (G, H) GBD degraded METTL3 through the ubiquitin‐proteasome pathway. Cells were treated with GBD with and without cycloheximide (G) or MG132 (H) for 2 h (I). GBD induced METTL3 ubiquitination. Cells were treated with GBD. METTL3 protein was immunoprecipitated with METTL3 antibody.

Finally, we investigated whether GBD exerted chondroprotective effects by influencing ATG7 m^6^A modification in chondrocyte cells. Consistent with the animal study, GBD treatment decreased the level of m^6^A and METTL3 expression (Figure 7A–C). GBD could also effectively reverse the decline of ATG7 mRNA as well as half‐life changes induced by IL‐1β (Figure 7D–F). Furthermore, we found that GBD significantly inhibited IL‐1β‐induced increase of m^6^A levels of ATG7 in chondrocyte cells, based on the MeRIP‐qPCR analysis (Figure 7G). To assess whether ATG7 m^6^A modification were required for chondroprotective effect of GBD, we constructed METTL3 mutant recombination plasmid for METTL3 overexpression (Figure 8A). Our data showed that METTL3 overexpression had little effect on the LC3II/LC3I ratio and ATG7 level when compared with the empty vector (Figure 8B–D). Nevertheless, overexpression of METTL3 significantly counteracted the enhanced autophagy as well as chondrocyte apoptosis inhibition (Figure 8E,F) induced by GBD, implying that GBD regulated chondrocyte autophagy and apoptosis through targeting ATG7 m^6^A modification in a METTL3‐dependent manner.

### 3.8 GBD promoted proteasome‐mediated ubiquitination degradation of METLL3

It is well known that proteasome‐dependent and lysosome‐dependent degradation are two important ways of protein degradation. In order to explore how GBD regulates the progression of OA, we sought to investigate the mechanism by which GBD regulates METLL3 degradation. Coincubation of specific lysosomal inhibitor cycloheximide displayed little effect on METLL3 downregulation (Figure 8G), whereas the specific proteasome inhibitor MG132 abolished the degradation of METLL3 by GBD (Figure 8H). Consistently, we also found that the METLL3 ubiquitination was markedly increased in the presence of GBD (Figure 8I).

## 4 DISCUSSION

KOA is a common joint disease that imposes huge social costs on society. The current conventional treatment for KOA such as NSAIDs could not directly protect cartilage against injuries, and cause kinds of adverse effects. Therefore, the use of natural ways, including herbal formulas, has garnered much interest for its effectiveness on KOA and absence of side effects.^26^ Herbal formula GBD has been widely used to ameliorate KOA progression, but has not been adequately studied. In this work, the anti‐osteoarthritic effect of GBD and its underlying mechanism were investigated. Osteoarthritis is characterized by articular cartilage degradation, subchondral osteosclerosis, osteophyte formation, and synovitis.^27^ The DMM mouse model is a common in vivo model to mimic the degenerative process during KOA.^28^ IL‐1β, as an inducer of OA microenvironment, has been recognized as a homogenous substance for establishing cellular KOA models.^29^ In the present study, both DMM‐induced KOA mice model and IL‐1β‐induced cellular model were established. The results showed that GBD could inhibit glycosaminoglycan loss and cartilage destruction, decreased the OARSI scores. The efficacy of high doses of GBD (34.96 g/kg/day) is close to that of positive drug diclofenac (10 mg/kg/day). Additionally, GBD increased protein expressions of ATG7 and LC3II, whereas decreased P62 levels via suppressing ATG7 m^6^A modification. GBD also restored the IL‐1β‐induced chondrocyte apoptosis and autophagy deficiency in vitro. METTL3 overexpression significantly counteracted the protective effect of GBD on chondrocyte autophagy. These findings highlight the anti‐degenerative effects of GBD against KOA, as might be mediated by promoting chondrocyte autophagy via METTL3‐dependent ATG7 m^6^A methylation.

Generally, KOA is characterized by progressive cartilage degradation, which is mainly caused by chondrocyte apoptosis or cytokine production.^30^ Cartilage is an integral part of the skeletal system, and contains chondrocytes which produce the ECM required for cartilage homeostasis.^31^ Proper cartilage development is essential to bone formation. The morphological and molecular features of chondrocyte apoptosis, including loss of nuclear volume and apoptotic bodies, have been detected in human KOA tissue specimens, and positively correlated with the severity of cartilage destruction.^17^ Currently, the apoptotic mechanisms in cartilage degeneration in KOA have been thoroughly explored.^32^, ^33^ It has been widely reported that transforming growth factor‐β(TGF‐β),^34^ fibroblast growth factors (FGFs),^35^ mitogen‐activated protein kinase, and Hippo/YAP signalling^36^ tightly modulate the cellular processes of chondrocytes. NO has been previously demonstrated to be the main player in chondrocyte apoptosis. In the presence of NO, a low level of ROS would induce chondrocyte apoptosis while high concentrations of ROS induce necrosis.^37^ Incubation of human articular chondrocytes with sodium nitroprusside (a chemical NO donor) increased gene expression of caspase‐3, caspase‐7, and downregulated bcl‐2 mRNA levels.^38^ On the other hand, chondrocyte apoptosis could also be induced in caspase‐independent manner, involving peroxynitrite‐induced mitochondrial dysfunction, calpains as well as calcium‐dependent cysteine proteases.^39^ Given the anti‐degenerative effects of GBD against KOA, we further demonstrated whether GBD inhibited chondrocyte apoptosis in articular cartilage. In animal study, TUNEL staining was conducted to assess the degree of chondrocyte apoptosis. We found that, the number of TUNEL‐positive cells significantly increased in the DMM mice but reduced after GBD treatment (Figure 3D,E). Furthermore, in IL‐1β‐stimulated chondrocytes, GBD treatment could remarkably reduce the cell apoptosis and increase cell viability, suggesting that GBD might produce chondroprotective effect through inhibiting chondrocyte apoptosis.

Next, we further investigated the action of GBD in regulating chondrocyte apoptosis. Our observation indicated that GBD may play an anti‐apoptotic role in chondrocytes by regulating autophagy. Autophagy is a conserved survival mechanism that protects cells, including chondrocytes, from apoptosis.^21^ During autophagy, cytosolic LC3‐I is conjugated to phosphatidylethanolamine and converted to LC3‐II.^40^ The ratio of LC3‐II/LC3‐I is widely considered as an indication of autophagy activation. Besides, autophagy is tightly regulated by autophagy‐related genes (ATGs) such as ATG7, LC3 and P62. P62 is an autophagy adaptor protein that recognizes unwanted cellular wastes, ATG7 coordinate the phagophore elongation, and Beclin1 is a core regulator of autophagosome biogenesis.^41^ Indeed, dysregulated autophagic activity has been widely reported during KOA development, and promotion of autophagic flux has been recognized as a promising therapeutic strategy for KOA.^42^, ^43^ Previous reports have shown that the protein and mRNA levels of LC3‐II, an autophagy marker, were decreased in articular cartilage of OA patients and in an animal OA model.^44^, ^45^ Activation of autophagy via ERK1/2 signalling pathways could inhibit apoptosis of chondrocytes.^46^ In this study, we assessed autophagy by measuring the protein abundance of LC3, P62 and ATG7 as well as the ratio of LC3‐II/LC3‐I. We found that autophagy was significantly inhibited in DMM mice and chondrocytes under the stimulation of IL‐1β (Figure 6D–H). GBD treatment effectively promoted the autophagic activity as evidenced by increased ATG7 and LC3‐II expression. Furthermore, inhibiting autophagy using chloroquine significantly reduced the anti‐apoptotic effect of GBD in chondrocytes (Figure S1), confirming that GBD protected chondrocyte form apoptosis by strengthening autophagy. Since tt has been reported that improving autophagy can effectively alleviate chondrocyte apoptosis and osteoarthritis,^14^ we speculate that GBD may play an anti‐KOA role by promoting autophagy.

Emerging as an abundant RNA modification in eukaryotic mRNA, m^6^A regulates a variety of biological processes such as gene expression, cell fate, and involves pathological and physiological events. m^6^A is triggered by methyltransferase (‘writers’, e.g., METTL3, METTL14), and removed by demethylase (‘erasers’, e.g., FTO, ALKBH5).^47^ m^6^A modifications affect the stability, selective splicing and translation of RNA, and thus affect apoptosis, autophagy and immune response.^48^ Recent studies found that disorder of m^6^A contributes to pathological bone diseases including KOA.^49^, ^50^ In addition to this, a recent work has reported that METTL3‐mediated m^6^A modification of ATG7 regulates autophagy to promote the OA progression.^25^ Given the promotion of GBD on ATG7 expression and chondrocyte autophagy, we further examined its regulation on m^6^A level of ATG7 and METTL3 expression. Our results m^6^A modification of ATG7 was significantly elevated in the model group, accompanied by increased METTL3 level. GBD effectively suppressed ATG7 m^6^A modification and METTL3 expression in vivo and in vitro studies (Figures 5 and 7). In addition, overexpression of METTL3 significantly eliminated the protective effects of GBD on chondrocytes against autophagy and apoptosis (Figure 8). These results suggested that chondroprotective effect exerted by GBD might be contributed by its inhibition on METTL3‐mediated ATG7 m6A modification.

## 5 CONCLUSION

This study demonstrated that herbal formula GBD possesses anti‐osteoarthritic effect in vitro and in vivo, via inhibiting METTL3‐mediated ATG7 m^6^A modification to promote autophagy. However, we acknowledge there are some limitations in our study. For example, METTL3‐knockout mice were not used to further verify the role of METTL3 in GBD's bone protective effect. The mechanism of how GBD promotes METTL3 ubiquitination needs to be further studied. More studies are still required to comprehensively demonstrate how GBD relieves the progression of KOA.

## AUTHOR CONTRIBUTIONS

**Longkang Cui:** Data curation (equal); investigation (equal); methodology (equal). **Gaobo Shen:** Software (equal); validation (equal); visualization (equal). **Yang Yu:** Data curation (equal); project administration (equal); visualization (equal). **Zheng Yan:** Conceptualization (equal); investigation (equal); methodology (equal). **Hanbing Zeng:** Data curation (equal); software (equal). **Xiaoang Ye:** Formal analysis (equal); software (equal). **Kuangying Xu:** Supervision (equal); validation (equal). **Chaojin Zhu:** Conceptualization (equal); supervision (equal). **Yanan Li:** Formal analysis (equal); investigation (equal). **Zhe Shen:** Project administration (equal); supervision (equal). **Bingbing Zhang:** Methodology (equal); writing – review and editing (equal). **Lianguo Wu:** Resources (lead); writing – review and editing (lead).

## CONFLICT OF INTEREST STATEMENT

The authors declare no conflict of interest.

## Supporting information


Figure S1.

## Data Availability

The data that support the findings of this study are available from the corresponding author upon reasonable request.

## References

[1] Hunter DJ , Bierma‐Zeinstra S . Osteoarthritis. Lancet. 2019;393(10182):1745‐1759. 31034380 10.1016/S0140-6736(19)30417-9

[2] Pelletier JP , Raynauld JP , Dorais M , et al. An international, multicentre, double‐blind, randomized study (DISSCO): effect of diacerein vs celecoxib on symptoms in knee osteoarthritis. Rheumatology. 2020;59(12):3858‐3868. 32521015 10.1093/rheumatology/keaa072 PMC7733720

[3] Zeng C , Zhang W , Doherty M , et al. Initial analgesic prescriptions for osteoarthritis in the United Kingdom, 2000–2016. Rheumatology. 2021;60(1):147‐159. 32594175 10.1093/rheumatology/keaa244

[4] Hamilton DF , Beard DJ , Barker KL , et al. Targeting rehabilitation to improve outcomes after total knee arthroplasty in patients at risk of poor outcomes: randomised controlled trial. BMJ. 2020;371:m3576. 33051212 10.1136/bmj.m3576 PMC7551789

[5] Bennell KL , Hinman RS . A review of the clinical evidence for exercise in osteoarthritis of the hip and knee. J Sci Med Sport. 2011;14(1):4‐9. 20851051 10.1016/j.jsams.2010.08.002

[6] Feng K , Chen Z , Pengcheng L , Zhang S , Wang X . Quercetin attenuates oxidative stress‐induced apoptosis via SIRT1/AMPK‐mediated inhibition of ER stress in rat chondrocytes and prevents the progression of osteoarthritis in a rat model. J Cell Physiol. 2019;234(10):18192‐18205. 30854676 10.1002/jcp.28452

[7] Xu K , He Y , Moqbel SAA , Zhou X , Wu L , Bao J . SIRT3 ameliorates osteoarthritis via regulating chondrocyte autophagy and apoptosis through the PI3K/Akt/mTOR pathway. Int J Biol Macromol. 2021;175:351‐360. 33556400 10.1016/j.ijbiomac.2021.02.029

[8] Xue JF , Shi ZM , Zou J , Li XL . Inhibition of PI3K/AKT/mTOR signaling pathway promotes autophagy of articular chondrocytes and attenuates inflammatory response in rats with osteoarthritis. Biomed Pharmacother. 2017;89:1252‐1261. 28320092 10.1016/j.biopha.2017.01.130

[9] Yu L , Chen Y , Tooze SA . Autophagy pathway: cellular and molecular mechanisms. Autophagy. 2018;14(2):207‐215. 28933638 10.1080/15548627.2017.1378838 PMC5902171

[10] Duan R , Xie H , Liu ZZ . The role of autophagy in osteoarthritis. Front Cell Dev Biol. 2020;8:608388. 33324654 10.3389/fcell.2020.608388 PMC7723985

[11] Feng L , Feng C , Wang CX , et al. Circulating microRNA let‐7e is decreased in knee osteoarthritis, accompanied by elevated apoptosis and reduced autophagy. Int J Mol Med. 2020;45(5):1464‐1476. 32323821 10.3892/ijmm.2020.4534 PMC7138275

[12] Lian WS , Ko JY , Wu RW , et al. MicroRNA‐128a represses chondrocyte autophagy and exacerbates knee osteoarthritis by disrupting Atg12. Cell Death Dis. 2018;9(9):919. 30206206 10.1038/s41419-018-0994-y PMC6134128

[13] Zheng W , Li X , Liu D , et al. Mechanical loading mitigates osteoarthritis symptoms by regulating endoplasmic reticulum stress and autophagy. FASEB J. 2019;33(3):4077‐4088. 30485126 10.1096/fj.201801851R PMC6404578

[14] Sun K , Guo Z , Zhang J , et al. Inhibition of TRADD ameliorates chondrocyte necroptosis and osteoarthritis by blocking RIPK1‐TAK1 pathway and restoring autophagy. Cell Death Dis. 2023;9(1):109. 10.1038/s41420-023-01406-0 PMC10066284 37002200

[15] Bao J , Chen Z , Xu L , Wu L , Xiong Y . Rapamycin protects chondrocytes against IL‐18‐induced apoptosis and ameliorates rat osteoarthritis. Aging. 2020;12(6):5152‐5167. 32182210 10.18632/aging.102937 PMC7138594

[16] Yang M , Jiang L , Wang Q , Chen H , Xu G . Traditional Chinese medicine for knee osteoarthritis: an overview of systematic review. PLoS One. 2017;12(12):e0189884. 29267324 10.1371/journal.pone.0189884 PMC5739454

[17] Musumeci G , Aiello FC , Szychlinska MA , Di Rosa M , Castrogiovanni P , Mobasheri A . Osteoarthritis in the XXIst century: risk factors and behaviours that influence disease onset and progression. Int J Mol Sci. 2015;16(3):6093‐6112. 25785564 10.3390/ijms16036093 PMC4394521

[18] He X , Wang L , Zhou X , et al. Effect of Gubi prescription on caveolin‐1 expression and phosphoinositide 3 kinase/protein kinase B and Fas signal pathways in rats with knee osteoarthritis. J Tradit Chin Med. 2020;40:224‐235. 32242388

[19] Zeng YF , Wang R , Bian Y , Chen WS , Peng L . Catalpol attenuates IL‐1β induced matrix catabolism, apoptosis and inflammation in rat chondrocytes and inhibits cartilage degeneration. Med Sci Monit. 2019;25:6649‐6659. 31484919 10.12659/MSM.916209 PMC6752111

[20] Weng X , Lin P , Liu F , et al. Achyranthes bidentata polysaccharides activate the Wnt/β‐catenin signaling pathway to promote chondrocyte proliferation. Int J Mol Med. 2014;34(4):1045‐1050. 25176272 10.3892/ijmm.2014.1869

[21] D'Adamo S , Cetrullo S , Guidotti S , et al. Spermidine rescues the deregulated autophagic response to oxidative stress of osteoarthritic chondrocytes. Free Radic Biol Med. 2020;153:159‐172. 32305648 10.1016/j.freeradbiomed.2020.03.029

[22] Caramés B , Hasegawa A , Taniguchi N , Miyaki S , Blanco FJ , Lotz M . Autophagy activation by rapamycin reduces severity of experimental osteoarthritis. Ann Rheum Dis. 2012;71(4):575‐581. 22084394 10.1136/annrheumdis-2011-200557 PMC3294168

[23] Duarte JH . Osteoarthritis: autophagy prevents age‐related OA. Nat Rev Rheumatol. 2015;11(12):683. 10.1038/nrrheum.2015.145 26481437

[24] Li YS , Zhang FJ , Zeng C , et al. Autophagy in osteoarthritis. Joint Bone Spine. 2016;83(2):143‐148. 26453105 10.1016/j.jbspin.2015.06.009

[25] Chen X , Gong W , Shao X , et al. METTL3‐mediated m(6)A modification of ATG7 regulates autophagy‐GATA4 axis to promote cellular senescence and osteoarthritis progression. Ann Rheum Dis. 2022;81(1):87‐99. 34706873 10.1136/annrheumdis-2021-221091

[26] Khanna D , Sethi G , Ahn KS , et al. Natural products as a gold mine for arthritis treatment. Curr Opin Pharmacol. 2007;7(3):344‐351. 17475558 10.1016/j.coph.2007.03.002

[27] Deng Y , Lu J , Li W , et al. Reciprocal inhibition of YAP/TAZ and NF‐κB regulates osteoarthritic cartilage degradation. Nat Commun. 2018;9(1):4564. 30385786 10.1038/s41467-018-07022-2 PMC6212432

[28] Fang H , Huang L , Welch I , et al. Early changes of articular cartilage and subchondral bone in the DMM mouse model of osteoarthritis. Sci Rep. 2018;8(1):2855. 29434267 10.1038/s41598-018-21184-5 PMC5809364

[29] Wu J , Ma L , Wu L , Jin Q . Wnt‐β‐catenin signaling pathway inhibition by sclerostin may protect against degradation in healthy but not osteoarthritic cartilage. Mol Med Rep. 2017;15(5):2423‐2432. 28259981 10.3892/mmr.2017.6278 PMC5428759

[30] Chan BY , Fuller ES , Russell AK , et al. Increased chondrocyte sclerostin may protect against cartilage degradation in osteoarthritis. Osteoarthr Cartil. 2011;19(7):874‐885. 10.1016/j.joca.2011.04.014 21619935

[31] Chijimatsu R , Saito T . Mechanisms of synovial joint and articular cartilage development. Cell Mol Life Sci. 2019;76:3939‐3952. 31201464 10.1007/s00018-019-03191-5 PMC11105481

[32] Musumeci G , Castrogiovanni P , Mazzone V , Szychlinska MA , Castorina S , Loreto C . Histochemistry as a unique approach for investigating normal and osteoarthritic cartilage. Eur J Histochem. 2014;58(2):2371. 24998926 10.4081/ejh.2014.2371 PMC4083326

[33] Thomas CM , Murray R , Sharif M . Chondrocyte apoptosis determined by caspase‐3 expression varies with fibronectin distribution in equine articular cartilage. Int J Rheum Dis. 2011;14(3):290‐297. 21816026 10.1111/j.1756-185X.2011.01627.x

[34] Wang TY , Chen D . Differential roles of TGF‐β signalling in joint tissues during osteoarthritis development. Ann Rheum Dis. 2016;75:e72. 27543413 10.1136/annrheumdis-2016-210312 PMC5207340

[35] Ornitz DM , Marie PJ . Fibroblast growth factor signaling in skeletal development and disease. Genes Dev. 2015;29:1463‐1486. 26220993 10.1101/gad.266551.115 PMC4526732

[36] Sun K , Guo J , Guo Z , et al. The roles of the hippo‐YAP signalling pathway in cartilage and osteoarthritis. Ageing Res Rev. 2023;90:102015. 37454824 10.1016/j.arr.2023.102015

[37] Del Carlo M Jr , Loeser RF . Nitric oxide‐mediated chondrocyte cell death requires the generation of additional reactive oxygen species. Arthritis Rheum. 2002;46(2):394‐403. 11840442 10.1002/art.10056

[38] Maneiro E , López‐Armada MJ , de Andres MC , et al. Effect of nitric oxide on mitochondrial respiratory activity of human articular chondrocytes. Ann Rheum Dis. 2005;64(3):388‐395. 15708893 10.1136/ard.2004.022152 PMC1755391

[39] Hwang HS , Kim HA . Chondrocyte apoptosis in the pathogenesis of osteoarthritis. Int J Mol Sci. 2015;16(11):26035‐26054. 26528972 10.3390/ijms161125943 PMC4661802

[40] Nakatogawa H . Mechanisms governing autophagosome biogenesis. Nat Rev Mol Cell Biol. 2020;21(8):439‐458. 32372019 10.1038/s41580-020-0241-0

[41] Yang H , Wen Y , Zhang M , et al. MTORC1 coordinates the autophagy and apoptosis signaling in articular chondrocytes in osteoarthritic temporomandibular joint. Autophagy. 2020;16(2):271‐288. 31007149 10.1080/15548627.2019.1606647 PMC6984599

[42] Sacitharan PK , Bou‐Gharios G , Edwards JR . SIRT1 directly activates autophagy in human chondrocytes. Cell Death Dis. 2020;6:41. 10.1038/s41420-020-0277-0 PMC7260231 32528730

[43] Tang Q , Zheng G , Feng Z , et al. Trehalose ameliorates oxidative stress‐mediated mitochondrial dysfunction and ER stress via selective autophagy stimulation and autophagic flux restoration in osteoarthritis development. Cell Death Dis. 2017;8(10):e3081. 28981117 10.1038/cddis.2017.453 PMC5680575

[44] Wu SY , Du YC , Yue CF . Sirt7 protects chondrocytes degeneration in osteoarthritis via autophagy activation. Eur Rev Med Pharmacol Sci. 2020;24(18):9246‐9255. 33015765 10.26355/eurrev_202009_23006

[45] Zhang Q , Lai S , Hou X , Cao W , Zhang Y , Zhang Z . Protective effects of PI3K/Akt signal pathway induced cell autophagy in rat knee joint cartilage injury. Am J Transl Res. 2018;10(3):762‐770. 29636866 PMC5883117

[46] Li X , Feng K , Li J , et al. Curcumin inhibits apoptosis of chondrocytes through activation ERK1/2 signaling pathways induced autophagy. Nutrients. 2017;9(4):414. 28430129 10.3390/nu9040414 PMC5409753

[47] He L , Li H , Wu A , Peng Y , Shu G , Yin G . Functions of N6‐methyladenosine and its role in cancer. Mol Cancer. 2019;18(1):176. 31801551 10.1186/s12943-019-1109-9 PMC6892141

[48] Liu Y , Yang Y , Lin Y , et al. N(6)‐methyladenosine‐modified circRNA RERE modulates osteoarthritis by regulating β‐catenin ubiquitination and degradation. Cell Prolif. 2023;56(1):e13297. 35733354 10.1111/cpr.13297 PMC9816929

[49] Dominissini D , Moshitch‐Moshkovitz S , Schwartz S , et al. Topology of the human and mouse m^6^A RNA methylomes revealed by m^6^A‐seq. Nature. 2012;485(7397):201‐206. 22575960 10.1038/nature11112

[50] Hu S , Shen C , Yao X , et al. m^6^A regulator‐mediated methylation modification patterns and immune microenvironment infiltration characterization in osteoarthritis. BMC Med Genet. 2022;15(1):273. 10.1186/s12920-022-01429-z PMC9805027 36585683

