# Supplementary material for: Gubi decoction mitigates knee osteoarthritis via promoting chondrocyte autophagy through METTL3‐mediated ATG7 m6A methylation
Source: J Cell Mol Med. 2024 Aug 20;28(16):e70019. doi: 10.1111/jcmm.70019 (PMC11335466; doi:10.1111/jcmm.70019)
Supplement: Supplementary file 1 — Figure S1. [file JCMM-28-e70019-s001.pdf]

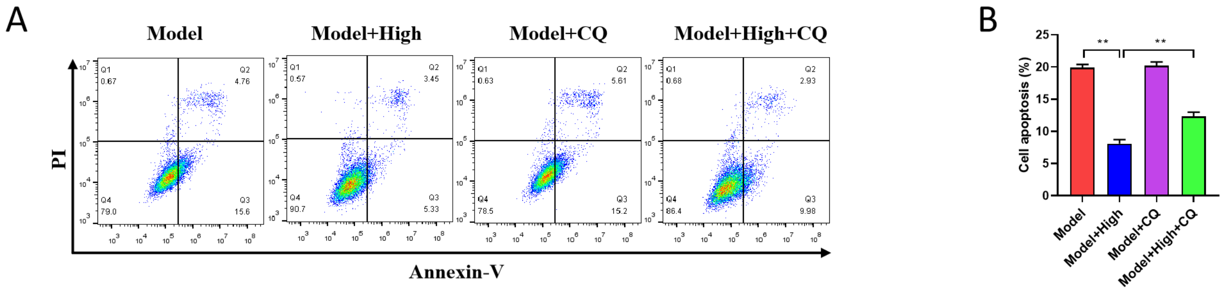

Supplementary Fig. 1

Supplementary Fig. 1. The effect of autophagy inhibitor chloroquine on the anti-apoptotic activity of GBD in chondrocyte cells detected by flow cytometry.
